# Intestinal immunoglobulins under microbial dysbiosis: implications in opioid-induced microbial dysbiosis

**DOI:** 10.3389/fmicb.2025.1580661

**Published:** 2025-04-14

**Authors:** Nicolas Vitari, Sabita Roy

**Affiliations:** ^1^Department of Microbiology and Immunology, University of Miami Miller School of Medicine, Miami, FL, United States; ^2^Department of Surgery, University of Miami Miller School of Medicine, Miami, FL, United States

**Keywords:** IgA, IgM, IgG, complement, opioids, intestinal immunity, microbiome

## Abstract

Intestinal immunoglobulins (Igs) maintain homeostasis between the microbiome and host. IgA facilitates microbial balance through a variety of increasingly well-described mechanisms. However, IgM and IgG have less defined intestinal functions but have the potential to activate clearance mechanisms such as the complement system and receptor-mediated bacterial killing. Very little is known regarding the role of Igs under microbial dysbiosis. In this review, we explore how Igs sculpt the intestinal microbiome and respond to microbial dysbiosis. We discuss how IgM, IgA, IgG, and complement individually maintain harmony with the microbiome and consider how these mechanisms could work in synergy. Finally, we explore using an opioid-induced microbial dysbiosis as a model to elucidate immediate changes in Ig-bacterial interactions.

## Introduction

Trillions of bacteria, viruses, and fungi live in the intestines of mammalian hosts. These organisms are collectively referred to as the microbiome. Under homeostatic conditions, the microbiome is essential for providing nutrients and protecting the host. The host is constantly exposed to the microbiome, which results in complex immune interactions to maintain homeostasis. Immunoglobulins (Igs) A, G, and M are primary mediators of intestinal homeostasis (Chen et al., 2020). Of all Igs, IgA is the most abundant at mucosal sites and is strongly induced by the microbiota, though recent work highlights the role of IgM and IgG in maintaining microbial homeostasis (Sterlin et al., 2020; Eriksen et al., 2023). IgM and IgG induce bacterial clearance via receptor-mediated killing and by activating the classical complement cascade. However, the roles of IgM and IgG in the intestinal microbiome are lesser understood. Additionally, an intestinal complement system independent of circulation was recently described (Wu et al., 2024). Here, we explore the individual roles of intestinal Igs and complement, and postulate on the synergistic implications of Igs and complement in maintaining microbial homeostasis. Ultimately, we discuss the role and therapeutic potential of Igs and complement in opioid-induced microbial dysbiosis.

## Basic intestinal immunoglobulin biology

### Structure of Igs

Igs are Y-shaped glycoproteins produced by B cells, plasma cells, and plasmablasts against pathogenic invaders. The Ig structure is made of two parts: the Fragment crystallizable (Fc) region and the Fragment antigen-binding (Fab) region. The Fc region determines the effector function of the Ig, while the Fab region is specific for antigen. There are 5 isotypes of Ig in mammals: IgA, IgM, IgG, IgE, and IgD.

Secreted IgM is the first antibody line of defense against pathogens and is produced by B cells prior to class switching to other isotypes. Secreted mouse IgM exists primarily in multimeric form, consisting of 5 IgM monomers connected via the Joining chain (J chain) to form pentamers (Brandtzaeg and Prydz, 1984). Due to this pentameric structure, IgM efficiently activates the complement system, and initiates receptor-mediated killing. Mouse IgM is present at high concentrations in circulation, and very low concentrations at mucosal surfaces (Hockenberry et al., 2023).

Following isotype switching to IgG, mice have four subtypes of IgG: IgG1, IgG2a/c, IgG2b, and IgG3. Secreted IgG are monomeric. Mouse and human IgG subtypes are distinct but share some similarities (Castro-Dopico and Clatworthy, 2019). IgGs have different abilities to activate complement or facilitate receptor-mediated killing based on the subtype (Wang et al., 2022; Bruhns and Jönsson, 2015). Like IgM, mouse IgG is present at high concentrations in circulation and low concentrations at mucosal surfaces.

IgA is the most abundant antibody produced by the body. At mucosal surfaces, the most common antibody isotype is IgA by a significant margin. Mice have one form of IgA, while humans have two—IgA1 and IgA2 (Steffen et al., 2020). Secreted IgA molecules exist in multimeric form, consisting of two IgA monomers connected via the J chain to form dimers (Kumar Bharathkar et al., 2020). However, mouse IgA is not able to activate complement or initiate receptor-mediated killing. Instead, the function of IgA is varied and context-dependent, as discussed later. Unlike IgM and IgG, IgA is present at relatively low levels in circulation, but extremely high concentrations at mucosal surfaces.

### IgM, IgA, and IgG transporters and receptors

IgM and IgA are multimeric, forming pentamers and dimers, respectively, connected by the J chain. Due to this multimeric structure, both IgM and IgA are transported across the epithelium by the poly-Ig receptor (pIgR) that is specific for the J chain (Kaetzel et al., 1991). By recognizing the J chain, pIgR specifically transports multimeric Igs (Brandtzaeg and Prydz, 1984). In the intestine, pIgR is expressed primarily on the basolateral portion of epithelial cells which allows IgA and IgM to be continuously transported unidirectionally from the lamina propria into the lumen (Apodaca et al., 1991; Mostov, 1991). Following transport, a portion of the pIgR transporter called the secretory component (SC) is released along with IgA or IgM (Tomasi et al., 1965). The combined complex of IgM or IgA with SC is commonly referred to as secretory IgM or IgA. SC increases the stability of secreted antibodies, reduces proteolytic degradation, and aids in recognition of microbes (Stadtmueller et al., 2016). SC can be secreted into the intestines independently of IgM or IgA and has the potential to impact the microbiome (Corthésy, 2013), but is not a focus of the current study and will not be discussed.

Both IgM and IgA bind to the Fcɑ/μ receptor (Fcɑ/μR), but this recognition is independent of the J chain (Yoo et al., 2011). IgM has a 10-fold higher affinity for Fcɑ/μR than IgA (Ghumra et al., 2009). Unlike pIgR, Fcɑ/μR is expressed by both hematopoietic and non-hematopoietic cells systemically (Liu et al., 2019). Binding of IgM or IgA to Fcɑ/μR results in systemic pro-inflammatory response and phagocytosis of the IgM-bound antigen (Liu et al., 2019).

Although there is no IgA-specific receptor identified in mice, the Fcμ receptor (FcμR) is specific for IgM. FcμR is expressed primarily by B cells in mice (Liu et al., 2019). FcμR plays an essential role in the differentiation, survival, and activation of B cells (Liu et al., 2019). IgM-bound antigens can be phagocytosed following FcμR recognition. Additionally, mice lacking the FcμR exhibit alterations in the maturation and differentiation of B cells, resulting in modulation of the humoral response (Liu et al., 2019).

There are four Fcγ receptors (FcγRI, FcγRIIB, FcγRIII, and FcγRIV) and one transporter specific for IgG in mice (Bruhns and Jönsson, 2015). IgG is transported across the intestinal epithelial barrier via the neonatal Fc receptor (FcRn) in a pH-dependent manner (Pyzik et al., 2015). FcRn is specific for the Fc portion of IgG with varying affinity in mice (Rath et al., 2013). FcRn is expressed on both the basolateral and apical sides of intestinal epithelial cells, which facilitates bidirectional transport of IgG (Dickinson et al., 1999). However, FcRn is also expressed on myeloid cells and plays an important role in identifying IgG immune complexes (Pyzik et al., 2023).

Fcγ receptors are expressed widely on myeloid, B, and natural killer cells throughout the body to facilitate clearance of opsonized microbes. Fcγ receptors have varying affinities for the four subtypes of mouse IgG (Bruhns and Jönsson, 2015). FcγRIIB and FcγRIII recognize IgG1, IgG2a/c and IgG2b, while FcγRI and FcγRIV recognize only IgG2a/c and IgG2b (Bruhns and Jönsson, 2015). There is conflicting evidence of IgG3 interacting with known mouse Fcγ receptors (Bruhns and Jönsson, 2015; Gavin et al., 1998; Saylor et al., 2010). In summary, binding to Fcγ receptors can lead to immune cell activation (FcγRI, FcγRIII, FcγRIV) or inhibition (FcγRIIB).

### Intestinal IgA dictates microbiome composition

**Figure 1:**
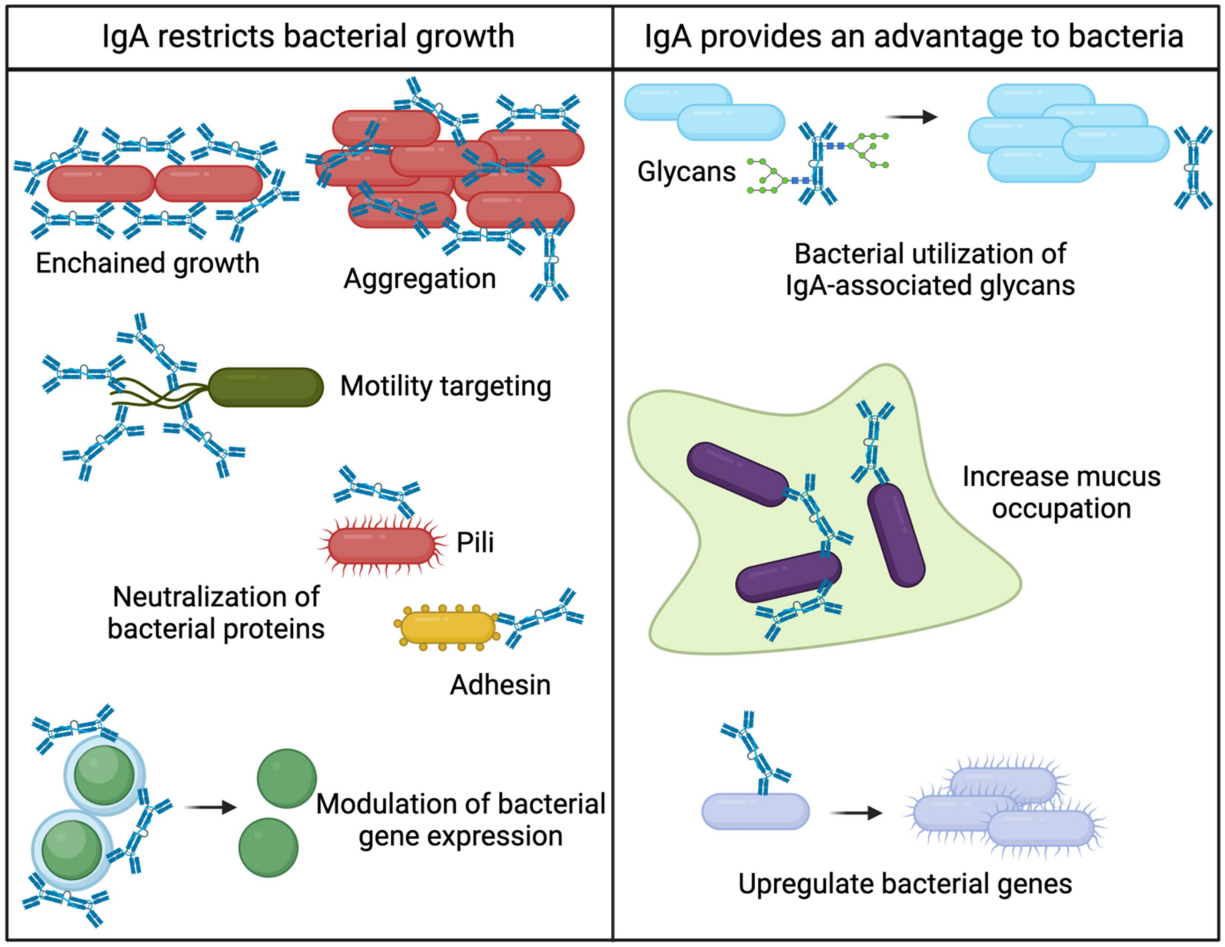
Bacterial fates of IgA binding. Intestinal IgA can both provide bacteria with a competitive advantage or restrict bacterial growth. Various mechanisms for restricting bacterial growth have been elucidated including 1. Enchained growth (Moor et al., 2017), 2. Aggregation (Pabst and Slack, 2020; Weis and Round, 2021; Michetti et al., 1992), 3. Motility targeting (Wold et al., 1990; Forbes et al., 2008; Cullender et al., 2013; Levinson et al., 2015), 4. Neutralization of bacterial proteins (Wold et al., 1990), and 5. Modulation of bacterial gene expression (Peterson et al., 2007; Nakajima et al., 2018). The mechanisms for IgA providing a competitive advantage to bacterial are 1. Bacterial utilization of IgA-associated glycans (Briliute et al., 2019; Mathias and Corthésy, 2011), 2. Increased mucus occupation (Steffen et al., 2020), and 3. Upregulation of bacterial genes (Nakajima et al., 2018).

Being the most abundant antibody produced by the body, the role of IgA has been extensively postulated. Many studies aim to address the functional role of IgA, and multiple lines of evidence conclude that the role of IgA is often dependent on context. Functional roles that have been assigned to IgA include facilitating colonization of bacteria, preventing microbial invasion, maintaining homeostasis with the microbiome, and selecting the composition of the microbiome. Subsequently, the functions of IgA have been summarized thoroughly (Chen et al., 2020; Sterlin et al., 2020; Hockenberry et al., 2023; Pabst and Slack, 2020; Weis and Round, 2021; Abokor et al., 2021). Though IgA is a master liaison between the microbiome and host, the phenotype for IgA deficiency is relatively mild, as IgM compensates to a certain degree in IgA-deficient settings (Catanzaro et al., 2019). However, IgA has been demonstrated to both limit and promote colonization of bacteria in a context-dependent manner, making IgA the master intermediary between host and the microbiome (Figure 1).

### Intestinal IgA limits bacterial growth

Many studies on IgA are conducted in the context of disease, since numerous chronic diseases result in microbial dysbiosis characterized by intestinal invasion of pathogenic bacteria (DeGruttola et al., 2016). Recently, it has been established that IgA is rarely sufficient for bacterial clearance but instead limits the overgrowth of pathogenic bacteria (Palm et al., 2014). This notion has been supported and is a currently accepted function of intestinal IgA during disease (Peterson et al., 2007). The primary mechanism for IgA limiting bacterial growth is broadly termed “immune exclusion” (Pabst and Slack, 2020; Weis and Round, 2021; Michetti et al., 1992). Immune exclusion refers to the aggregation of bacteria in such a way that bacteria are confined, and passage through the epithelial barrier is prevented. As such, pathogenic bacteria that elicit potent immune responses are generally highly targeted by IgA (Palm et al., 2014). By confining bacteria into aggregates, IgA can prevent bacterial replication and curtail bacteria-bacteria interactions. An elegant study demonstrated clearance of pathogenic IgA-bound bacteria via a specific aggregation process coined “enchained growth” (Moor et al., 2017). IgA binds rapidly dividing bacteria and prevents their separation, which results in a chain of bacteria that are unable to disseminate throughout the intestine. Immune exclusion and bacterial aggregation by IgA therefore result in control of intestinal bacteria without activating intestinal inflammation.

IgA binding to bacteria also alters gene expression, motility, and adhesion of bacteria, which presents additional mechanisms for maintaining intestinal homeostasis. Intestinal IgA neutralizes pili (Wold et al., 1990) and flagella (Forbes et al., 2008; Cullender et al., 2013; Levinson et al., 2015) of pathogens that could limit their ability to colonize. By minimizing bacterial interactions and motility, IgA could prevent biofilm formation thereby reducing colonization efficacy (Nakahashi-Ouchida et al., 2022). In addition, IgA binding alters the expression of essential bacterial enzymes that could result in slowing of bacterial growth (Peterson et al., 2007; Nakajima et al., 2018). Together, there is a rich literature on how IgA binding results in multiple mechanisms that selectively limit the growth of pathogenic bacteria. Importantly, IgA maintains intestinal homeostasis through these mechanisms, and the result of limiting pathogenic bacterial growth is facilitating maintenance of the normal flora (Moor et al., 2017).

### Intestinal IgA provides a competitive advantage to microbes

Novel mechanisms for IgA promoting bacterial growth have been shown *in vitro*, but many proposed mechanisms have not been validated *in vivo*. However, recent *in vivo* studies show that IgA can selectively promote the growth of certain bacteria. This notion is further supported by the repeated observations that commensal bacteria are often highly coated with IgA (Palm et al., 2014; Scheithauer et al., 2021; Vitari et al., 2024; Huus et al., 2020), and that beneficial commensal bacteria have decreased IgA binding during malnutrition (Kau et al., 2015). IgA has been demonstrated to enhance the anchoring ability of bacteria to the mucosa, which provides a competitive advantage for colonization (Donaldson et al., 2018). In addition, IgA is highly decorated with glycans (Steffen et al., 2020). Intestinal bacteria can digest IgA-derived glycans, which could promote their survival (Briliute et al., 2019; Mathias and Corthésy, 2011). It was recently demonstrated that bacteria could use IgA-derived glycans for survival and expansion in the absence of other food sources (Briliute et al., 2019). However, glycobiology is a notoriously understudied aspect of IgA biology (Pabst and Slack, 2020), but remains a promising direction for how IgA mediates intestinal homeostasis.

During inflammatory disease, IgA is produced against expanding pathogenic bacteria, which has led to many studies focusing on how IgA neutralizes pathogens. Therefore, much of the literature investigates intestinal IgA in an inflammatory setting. However, probiotic strains of bacteria induce an intestinal IgA response in the absence of inflammation (Huus et al., 2020; Rodrigues et al., 2000; Takeuchi and Ohno, 2022). IgA production induced by beneficial commensals indicates that IgA facilitates survival of beneficial commensals. It is necessary for studies to demonstrate the mechanisms for how IgA promotes survival of beneficial commensals. This line of investigation could result in novel methods for encouraging growth of beneficial commensals, which would prevent or limit the expansion of pathogenic bacteria.

### Intestinal IgM and IgG during homeostasis and microbial dysbiosis

Despite their ability to elicit complement- and Fc receptor-mediated killing of bacteria, the intestinal roles of IgM and IgG are not extensively studied. Maternal IgM and IgG help train the early intestinal immune system and are present at high concentrations during early life but quickly dissipate during maturation (Koch et al., 2016). IgM- and IgG-secreting cells are present at low numbers in comparison to IgA, and the concentration of intestinal IgM and IgG is low during homeostasis (Hockenberry et al., 2023). However, recent work is beginning to outline how IgM and IgG work in concert with IgA to maintain homeostasis during disease (Chen et al., 2020; Eriksen et al., 2023; Fadlallah et al., 2019; Bäumler and Sperandio, 2016). The functional roles of IgM and IgG have been identified in multiple disease states such as inflammatory bowel diseases (IBD), enteric infections, and Ig deficiency.

### IgM

Intestinal IgM seemingly plays two distinct roles: safeguarding microbial homeostasis and protecting against invading microbes. With the recently developed ability to sequence Ig-bound bacteria, it was discovered that IgM binds a small set of bacteria distinct from IgA under homeostatic conditions (Magri et al., 2017). In IgA-deficient settings, intestinal IgM binds to bacteria to partially compensate for the lack of IgA (Fadlallah et al., 2018). Work from the Gorochov group reveals that *Actinobacteria* are spared by IgM binding in IgA-deficient settings but are absent in IgM- and IgA-deficient settings (Fadlallah et al., 2018). This work suggests that IgM binding promotes bacterial diversity, supporting the notion that intestinal IgM is essential to maintaining microbial homeostasis. Unlike humans, there is very little IgM binding to bacteria at homeostasis in mice (Magri et al., 2017), despite free IgM being present at low concentrations in the luminal content (Lycke et al., 1999). However, IgM is unable to target all bacterial taxa targeted by IgA, which leads to overgrowth of certain bacteria and increases the host’s susceptibility to enteric infections in IgA-deficient settings.

**Figure 2:**
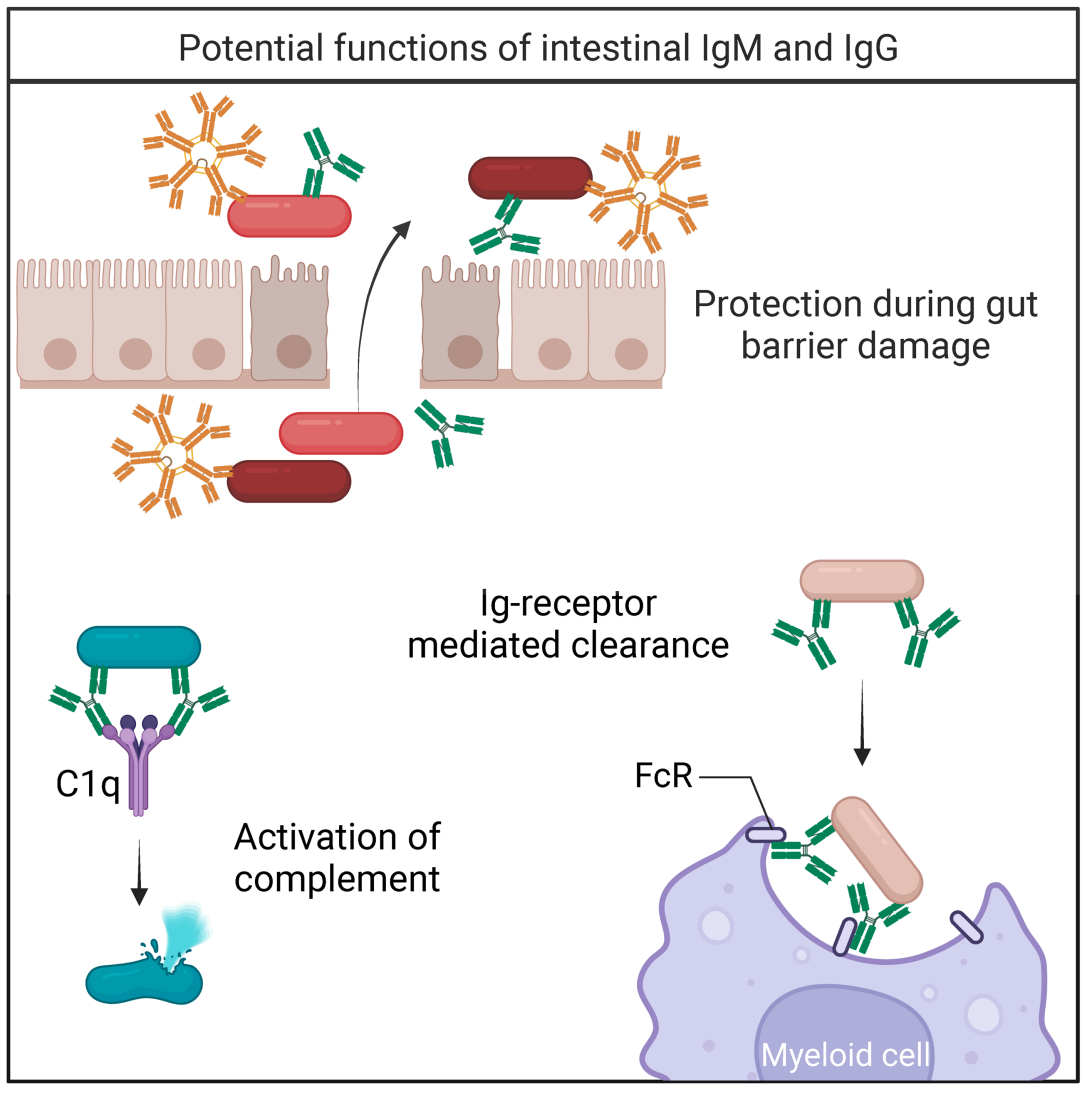
Bacterial fates of IgM/IgG binding. During gut barrier damage and inflammation, IgM and IgG protect against translocating bacteria (Kirkland et al., 2012; Masu et al., 2021). Upon binding to IgM or IgG, bacteria can be cleared by multiple mechanisms including 1. Activation of the complement cascade (Dunkelberger and Song, 2010), and 2. Receptor-mediated clearance (Nesargikar et al., 2012).

IgM is present at low quantities in the luminal content in healthy individuals and mice but increases during intestinal inflammation to protect against damage (Kirkland et al., 2012). Indeed, the concentration of IgM and frequency of IgM-bound bacteria increases during IBD (Masu et al., 2021). Importantly, activation of the classical complement system downstream of IgM was essential to protecting mice from fatal bacterial translocation during DSS-induced colitis (Kirkland et al., 2012). In addition to complement activation, IgM prevents the translocation of intestinal pathogens by agglutinating intestinal bacteria (Bioley et al., 2017). Together, these studies demonstrate that intestinal IgM promotes microbial homeostasis and protects against invading microbes (Figure 2).

### IgG

Intestinal IgG plays a protective role during disease, though the homeostatic function of IgG is still being established. A subset of the microbiome is coated by IgG in healthy individuals and mice, suggesting that intestinal IgG may contribute to bacterial composition (Zeng et al., 2016; van der Waaij et al., 2004). Alternatively, a substantial body of work describes the protective function of intestinal IgG during microbial dysbiosis and infection (Sterlin et al., 2020). Protective intestinal IgG was first described following gut barrier disruption during IBD (van der Waaij et al., 2004). Since then, multiple studies describe the repertoire of circulating commensal reactive IgGs (Fadlallah et al., 2019; Zeng et al., 2016; Haas et al., 2011). Systemic anti-commensal IgG and IgG coating of intestinal bacteria markedly increase during IBD, as well as the number of intestinal IgG-secreting cells (van der Waaij et al., 2004; Vujkovic-Cvijin et al., 2022). The molecular mechanisms for systemic commensal-reactive IgG are yet to be defined, but it is currently thought that commensal-specific IgG responses are generated routinely in barrier disruption events (Sterlin et al., 2020).

IgG-deficient humans are more prone to intestinal infections, suggesting that intestinal IgG is essential in protecting against certain pathogens even with the presence of IgA (Oksenhendler et al., 2008). Intestinal IgG-secreting cells are present in both humans and mice (Castro-Dopico and Clatworthy, 2019; Verma et al., 2024). Particularly, IgG2b- and IgG3-secreting cells have been identified in the Peyer’s Patches and mesenteric lymph nodes of mice (Koch et al., 2016). Mouse IgG2b and IgG3 essential for immunity toward repeated polysaccharide antigens (Greenspan and Cooper, 1993). Due to its’ oligomerization potential, mouse IgG3 is known to activate complement strongly (Klaus et al., 1979). However, low concentrations of mouse IgG2b also fixes complement efficiently (Klaus et al., 1979). IgG has strong effector functions and present a plausible mechanism for maintaining microbial homeostasis (Figure 2).

### Intestinal complement in microbial homeostasis

IgM or IgG binding to an antigen activates the classical complement pathway (Dunkelberger and Song, 2010). Following Ig-binding, microbes are either directly killed by myeloid cells or the rest of the complement cascade is activated for bacterial clearance (Nesargikar et al., 2012). IgM pentamers fix complement extremely efficiently, while IgG activates complement in a less efficient manner (Klaus et al., 1979; Czajkowsky and Shao, 2009). Following Ig recognition of an antigen, complement component 1q (C1q) combines with C1r and C1s to form the C1 complex (Dunkelberger and Song, 2010). The C1 complex initiates the classical complement cascade by cleaving C2 and C4, which ultimately makes C3 convertase that cleaves C3. There are two other pathways that activate complement—the alternative and mannose binding lectin (MBL) pathways—which all converge on C3. The alternative pathway is initiated by the spontaneous hydrolysis of C3, while the lectin pathway is initiated by MBL and recruitment of MBL-associated serine protease (MASP) 1 and MASP2. Following C3 conversion, complement components C5-C9 are recruited to eventually formulate the membrane attack complex, which forms pores in the bacterial surface resulting in bacterial killing. Alternatively, C3b and C5b are opsonins that coat pathogens making them more susceptible to being ingested by phagocytes, such as macrophages and neutrophils (Esser, 1994). Unlike the complement system that is highly dominant in circulation and provides immediate protection against systemic invaders, the intestinal roles of complement are still not well elucidated.

**Figure 3:**
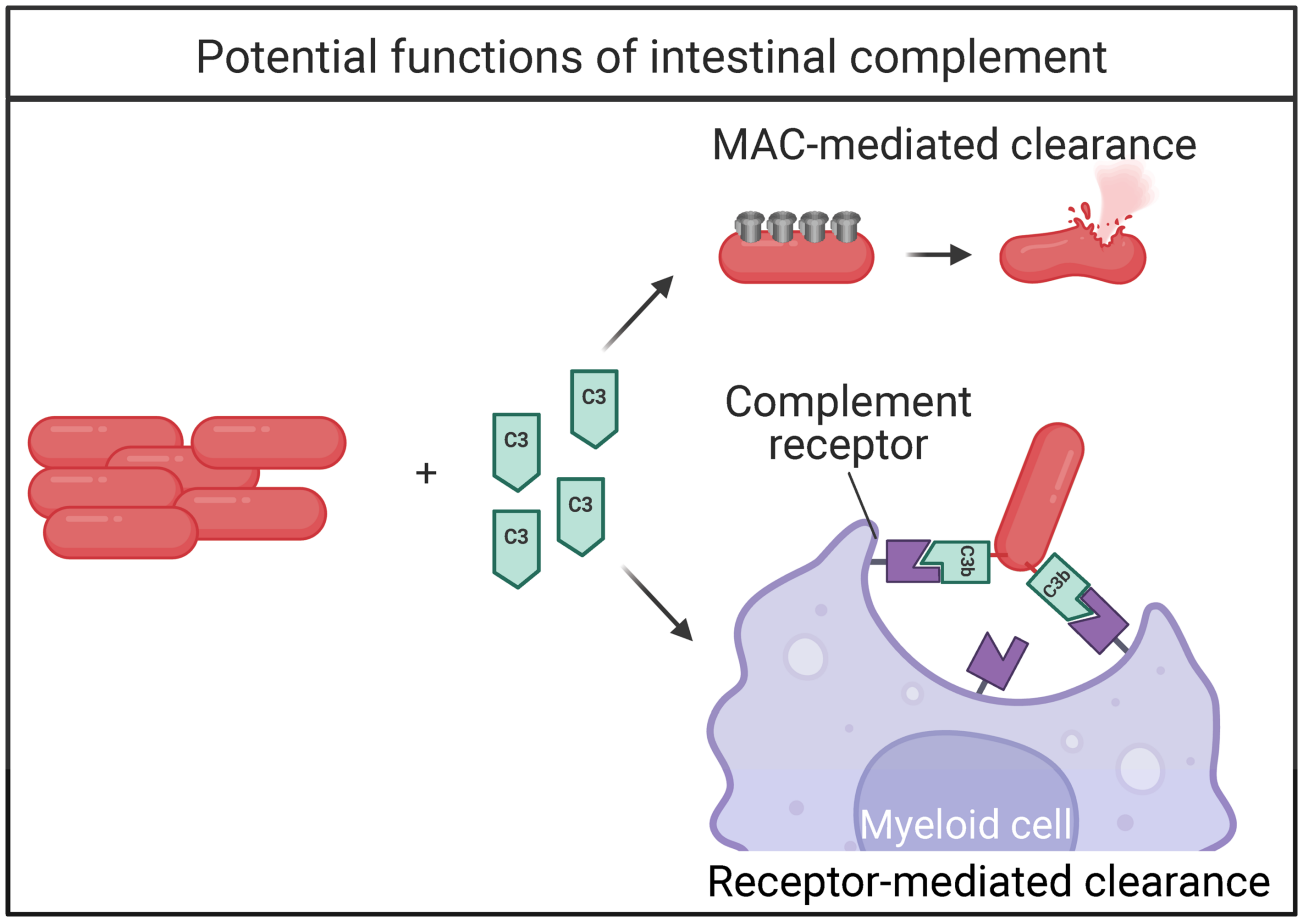
Bacterial fates of intestinal complement activation. When the complement cascade is activated, bacteria can be directly killed via 1. recruitment of membrane attack complex (MAC) proteins, or 2. complement-receptor mediated clearance by myeloid cells.

Although the complement system has been identified in the intestine many years ago, its relationship with the gut microbiome was unknown until recently (Halstensen et al., 1989). During intestinal inflammation, the transcription and amount of complement protein increases (Chen et al., 2011). An intestinal complement system independent of circulation was described to be dependent on the microbiome and facilitate clearance of pathogenic bacteria while sparing commensals (Wu et al., 2024). The intestinal complement system is highly dependent on complement component C3. Importantly, Wu and colleagues demonstrate local production of complement components, primarily C1-C4. Intestinal expression of C5-C9 was low, suggesting that the membrane attack complex does not form. Instead, the authors elegantly describe C3-mediated clearance of pathogenic bacteria by myeloid cells (Figure 3; Wu et al., 2024). Intestinal C3 was present in both mice and humans, and the concentration of C3 positively correlated with the relative abundance of certain commensal bacteria *Prevotella* and *Enterococcus.* Further, C3 expression was directly stimulated by both *Prevotella* and *Enterococcus in vitro*, and colonization with segmented filamentous bacteria (SFB) increased the concentration of fecal C3 in germ-free (GF) mice (Wu et al., 2024). This work represents a major advancement in the field and is an exciting new mechanism for immune maintenance of the microbiome. Further, this work implies that complement may play an important role during microbial dysbiosis.

### Impact of opioids on antibody responses

Since their widespread clinical use beginning in the 1800s (Jones et al., 2018), opioids are the most effective treatment for moderate to severe pain (Rosenblum et al., 2008). Morphine, alongside fentanyl, oxycodone, hydrocodone and codeine, is one of the most widely used prescription opioids in the United States. Widely known for its’ addictive and abuse potential, morphine has many additional adverse effects such as constipation, increase risk of sepsis, immune modulation, and has recently been shown to cause intestinal microbial dysbiosis (Banerjee et al., 2016; Meng et al., 2013; Meng et al., 2015; Ninkovic et al., 2016; Sharma et al., 2020).

There are three opioid receptors expressed on immune cells: μ (Mu opioid receptor) (MOR), k (KOR), and d (DOR) (Evans et al., 1992; Simonin et al., 1995; Mestek et al., 1995). Each opioid receptor has their own endogenous ligand with significant cross-reactivity with other opioid receptors. Those endogenous ligands are β-endorphin, dynorphin, and methionine-enkephalin for MOR, KOR, and DOR, respectively (Valentino and Volkow, 2018). The analgesic properties of opioids as well as respiratory depression and constipation, are downstream of MOR binding (Machelska and Celik, 2018; Yaksh, 1997). Morphine binds to MOR and is used clinically to alleviate moderate to severe pain. MOR is a seven transmembrane-domain guanine nucleotide-binding protein (G protein) coupled receptor (GPCR) consisting of three protein components (α, β, γ) bound to GDP (Mestek et al., 1995). Binding of a mu agonist to MOR will replace the GDP with a GTP, releasing Gα-GTP and βγ subunits. These subunits cause changes to the cell, including activation of the MAPK pathway, phospholipase C, adenylyl cyclase, and potassium and calcium channels (Standifer and Pasternak, 1997). Cellular changes after mu opioid receptor binding ultimately result in cellular dysfunction, aberrant activation, senescence, or apoptosis.

Opioid use is associated with many psychiatric and physical co-morbidities in humans, including anxiety, depression, chronic pain, cancer, pancreatitis, and heart disease (Levis et al., 2021; Jalodia et al., 2022; Garland et al., 2013; Schulte and Hser, 2014; Kesh et al., 2024). Further, opioid use exacerbates the progression of many chronic and infectious diseases (Sharma et al., 2020; Schwetz et al., 2019). Therefore, it was postulated that opioids have profound impacts on the immune system. It was found that people who inject drugs are immunocompromised and more susceptible to infection (Edelman et al., 2019). Indeed, the immunomodulatory effects of opioids have been explored extensively in humans, mice, and rats (Eisenstein, 2019; Odunayo et al., 2010). *In vitro* and *ex vivo* experiments have concluded that direct exposure to opioids results in a systemic decrease in the effector function of many immune cells (Liang et al., 2016). However, recent work reveals that opioids induce intestinal inflammation and exacerbate intestinal disease (Meng et al., 2013; Sharma et al., 2020). These studies suggest that opioids depress and activate immune function in a context-dependent manner.

Opioids were first reported to reduce the antibody response in humans and mice in 1975 (Lefkowitz, 1975). Since then, studies focusing primarily on cultured splenocytes and immune cells isolated from circulation have revealed extensive modulation of antibody responses by opioids. Since the initial report, it is now understood that opioids decrease multiple aspects of the antibody response, including antibody secretion, activation, and proliferation of B cells (Bussiere et al., 1993; Thomas and House, 1995; Bryant et al., 1988). These studies pre-date the discovery that opioids induce microbial dysbiosis, and the importance of intestinal Igs in maintaining microbial homeostasis. Therefore, the intestinal antibody response to opioids needs to be revisited.

### Intestinal antibody responses during morphine-induced microbial dysbiosis

The impact of opioids on the intestinal antibody response has been explored. Multiple reports conclude that opioids inhibit the intestinal IgA and IgG response to cholera toxin (Dinari et al., 1989; Peng et al., 2001; Carr et al., 1990). Importantly, these studies employ experimental approaches over a period of 7–22 days and were conducted prior to the development of tools to probe Ig-bacterial dynamics. A landmark study from the Roy laboratory in 2016 showed for the first time that opioids impact the intestinal microbiome within 24 h (Banerjee et al., 2016). Subsequently, multiple opioids have been demonstrated to induce microbial dysbiosis at multiple time points in mice and humans (Vitari et al., 2024; Sharma et al., 2020; Acharya et al., 2017; Jalodia et al., 2022; Kolli et al., 2023; Meng et al., 2023; Zhang et al., 2019). Since this observation, multiple inflammatory processes originally reported to be downregulated with opioid use are upregulated in the intestines (Meng et al., 2015; Jalodia et al., 2022). Importantly, the recent intestinal investigations are focused on early time points of opioid use, which were overlooked in initial studies.

Since intestinal antibodies maintain microbial homeostasis, recent investigations aimed to outline how opioid-induced microbial dysbiosis alters this homeostasis. It was found that morphine-induced microbial dysbiosis disrupts IgA-bacterial homeostasis in the ileal luminal content within 24 h, which causes increased concentration of unbound IgA and a corresponding decrease in the frequency of IgA bound bacteria. However, TLR2 signaling between 24 and 48 h of morphine treatment resulted in IgA targeting of gram-positive bacteria that persists through 72 h. There was a decrease in the IgA indices of bacteria previously described to be commensal at both 24 and 48 h of morphine treatment. At 48 h of morphine treatment, both commensal and potentially pathogenic gram-positive bacteria had increased IgA indices, which supports a dual role for IgA in the context of morphine treatment. Previous reports show that intestinal CD11b-expressing IgA plasma cells are microbiome-dependent and are generated in a TLR-dependent manner (Fu et al., 2021; Gao et al., 2022; Kunisawa et al., 2013). We report increased expression of CD11b on intestinal IgA^+^ B cells, further supporting TLR involvement (Vitari et al., 2024). These findings indicate that opioid-induced microbial dysbiosis alters intestinal B cells, resulting in rapid IgA targeting of bacteria.

During opioid use, there is evidence for intestinal upregulation of complement genes (Choi et al., 2022). Therefore, we investigated how intestinal complement proteins were altered during morphine-induced microbial dysbiosis. We reported that morphine treatment increases expression of classical complement genes in the intestinal tissue with a corresponding increase in the concentration of both C1q and C3 in the ileal luminal content. Importantly, the concentration of IgM and IgG in the ileal luminal content increases alongside complement proteins. Microbiome depletion abrogated the increased concentration of IgM, IgG, and complement proteins, demonstrating that the changes are dependent on the microbiome. Paired with the recent observation that morphine induces intestinal myeloid cell infiltration (Jalodia et al., 2022), these studies reveal a potential function for intestinal activation of the classical complement system capable of responding to microbial dysbiosis. Taken together, morphine induces rapid microbial dysbiosis that causes a profound and immediate intestinal antibody response. Further, morphine treatment presents an excellent model for studying Ig dynamics at the onset of microbial dysbiosis, with the potential to gain insights on how Igs work together with complement to restore homeostasis.

While these studies focus on the microbial shifts with opioid use, opioids are known to alter TLR signaling directly (Zhang et al., 2020; Hutchinson et al., 2010; Watkins et al., 2005). TLR signaling influences production of immunoglobulins, and antibody-secreting cells express TLRs (Genestier et al., 2007; Dorner et al., 2009). Therefore, opioids may impact immunoglobulin production directly, leading to rapid changes. Together with the shift in microbiome during opioid treatment, opioids could directly and indirectly influence antibody-secreting cells via TLRs.

### Questions and perspectives

The role of antibodies in intestinal homeostasis has been unfolding for decades but is yet to be fully understood. Sequencing Ig-bound bacteria has revolutionized our ability to measure IgA, IgG, and IgM in the maintenance of microbial homeostasis. Now, it is appreciated that complement is important for maintaining intestinal harmony as well. However, the synergistic and therapeutic aspects of intestinal immunoglobulins and complement are still being explored.

### What is the fate of bacteria bound to multiple Ig isotypes?

**Figure 4:**
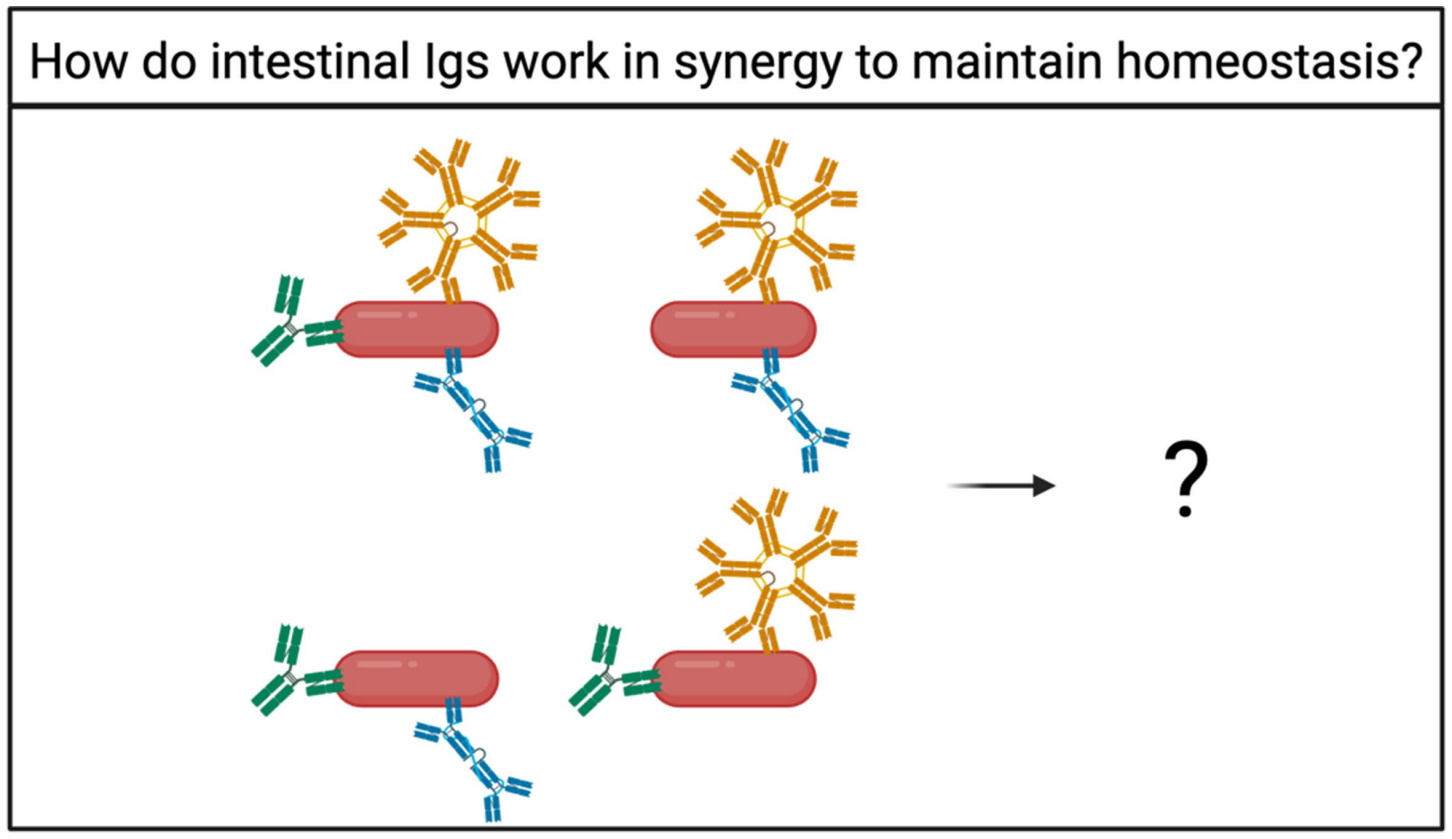
How do intestinal Igs work together to facilitate microbial maintenance? Bacteria can be bound by multiple Ig isotypes at once (Chen et al., 2020; Eriksen et al., 2023; Catanzaro et al., 2019). However, the fate of bacteria bound by multiple Ig isotypes is unknown.

Many studies narrow their scope by focusing on one Ig at a time, and most studies rarely mention other Ig isotypes. However, there is an emerging trend in recent literature that intestinal bacteria can be bound to IgA, IgM, or IgG individually or in combination (Chen et al., 2020; Eriksen et al., 2023; Catanzaro et al., 2019). The functional significance of bacteria binding to multiple Igs is largely unknown but could provide important answers on how homeostasis is maintained (Figure 4).

### How is the specificity of intestinal complement-mediated bacterial clearance determined?

**Figure 5:**
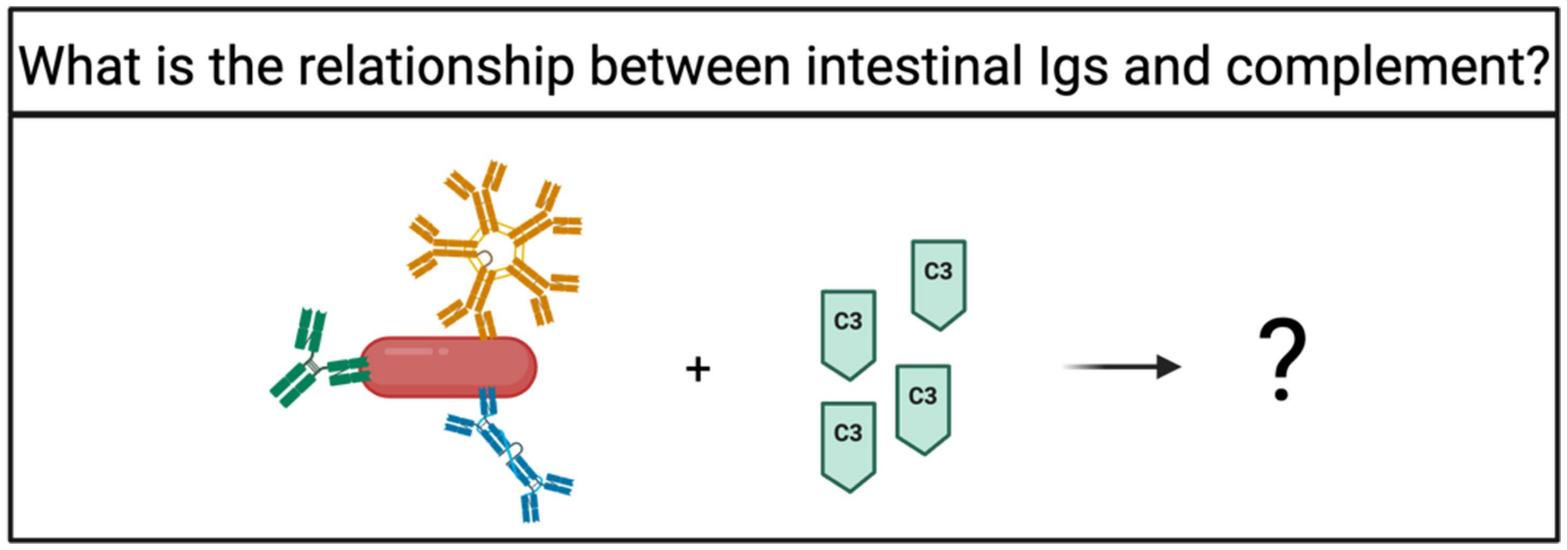
How is complement-mediated clearance of intestinal bacteria determined? Intestinal complement specifically controls the overgrowth of pathogenic bacteria (Wu et al., 2024), but the mechanism underlying that specificity is unknown. Since IgM and IgG activate complement, Ig binding to bacteria could account for the specific clearance of pathogens, however that remains undetermined.

Intestinal complement was described to selectively clear pathogenic bacteria while sparing commensal taxa, but the mechanism for this has not been elucidated. The concentration of C3 positively correlated with the relative abundance of *Prevotella* and *Enterococcus* in human microbiota, and colonization of GF mice with SFB induced a measurable increase in C3 concentration (Wu et al., 2024). Importantly, *Prevotella*, *Enterococcus*, and SFB are known to be highly targeted by Igs (Palm et al., 2014; Vitari et al., 2024; Larsen, 2017). Thus, it is plausible that there is a relationship between C3-mediated clearance of bacteria and Ig targeting (Figure 5). Are the determinants for intestinal complement generation the same for Ig production? Do intestinal Igs mark bacteria for complement-mediated clearance?

### Therapeutic implications: could intestinal antibodies and complement be used to mitigate opioid-induced microbial dysbiosis?

Due to it is stability, the idea of using IgA as a therapeutic for microbial dysbiosis and to limit the growth of pathogenic intestinal bacteria has been postulated (Wallace et al., 2020; Shinkura, 2021). Opioid-induced microbial dysbiosis is characterized by expansion of potentially pathogenic gram-positive bacteria including *Enterococcus*. Additionally, the concentration of intestinal complement is associated with the relative abundance of certain bacteria (Wu et al., 2024). Therefore, it is plausible that Igs and complement could be a therapeutic target to enhance the clearance of gram-positive bacteria or prevent their expansion during microbial dysbiosis. Could antigen-specific or complement-activating antibodies attenuate microbial dysbiosis during opioid use? Could these principles be extended to microbial dysbiosis associated with other diseases?

Due to recent technological advancements, the function of intestinal IgM and IgG are now being appreciated, and the role of intestinal IgA is now becoming well-understood. Now, the synergistic and therapeutic potential of intestinal Igs is emerging. Paired with the recently described intestinal complement system, it is plausible that further investigations could yield therapeutic advancements.
